# Early effect of oral administration of omeprazole with mosapride as compared with those of omeprazole alone on the intragastric pH

**DOI:** 10.1186/1471-230X-12-25

**Published:** 2012-03-26

**Authors:** Hiroshi Iida, Masahiko Inamori, Tetsuro Fujii, Yusuke Sekino, Hiroki Endo, Kunihiro Hosono, Takashi Nonaka, Tomoko Koide, Hirokazu Takahashi, Masato Yoneda, Ayumu Goto, Yasunobu Abe, Noritoshi Kobayashi, Hiroyuki Kirikoshi, Kensuke Kubota, Satoru Saito, Eiji Gotoh, Shin Maeda, Atsushi Nakajima

**Affiliations:** 1 Gastroenterology Division, Yokohama City University School of Medicine, 3-9 Fukuura, Kanazawa-ku, Yokohama, Japan; 2 Office of Postgraduate Medical Education, Yokohama City University Hospital, 3-9 Fukuura, Kanazawa-ku, Yokohama, Japan; 3 Department of Medical Education, Yokohama City University School of Medicine, 3-9 Fukuura, Kanazawa-ku, Yokohama, Japan

**Keywords:** Intragastric acidity, Omeprazole, Mosapride

## Abstract

**Background:**

The ideal medication for acid-related diseases should have a rapid onset of action to promote hemostasis and cause efficient resolution of symptoms. The aim of our study was to comparatively investigate the inhibitory effect on gastric acid secretion of a single oral administration of omeprazole plus mosapride with that of omeprazole alone.

**Methods:**

Ten Helicobacter pylori-negative male subjects participated in this randomized, two-way crossover study. Intragastric pH was monitored continuously for 6 hours after a single oral administration of omeprazole 20 mg or that of omeprazole 20 mg plus mosapride 5 mg (the omeprazole being administered one hour after the mosapride). Each administration was separated by a 7-days washout period.

**Results:**

The average pH during the 6-hour period after administration of omeprazole 20 mg plus mosapride 5 mg was higher than that after administration of omeprazole 20 mg alone (median: 3.22 versus 4.21, respectively; *p *= 0.0247).

**Conclusions:**

In H. pylori -negative healthy male subjects, an oral dose of omeprazole 20 mg plus mosapride 5 mg increased the intragastric pH more rapidly than omeprazole 20 mg alone.

## Background

The ideal medication for gastric acid-related diseases, especially for conditions like hemorrhagic gastric ulcers and stress-related gastric bleeding, should have a rapid onset of action to lower the intragastric acidity, because in vitro studies have shown that blood coagulation and platelet aggregation are abolished at pH less than 5.4 [1]. Medication for on-demand treatment should also have a rapid onset of action to ensure that the symptoms are controlled. Multiple agents, including antacids, histamine H2 receptor antagonists (H2RA) and proton pump inhibitors (PPI), are currently available for the treatment of these diseases. PPIs are the most potent inhibitors of gastric acid secretion when used regularly [2]. Our previous study demonstrated that administration of H2RA plus mosapride (orally) increased the intragastric pH more rapidly than that of H2RA alone [3]. However, no study has yet examined whether administration of PPI plus mosapride (orally) may also produce a more rapid increase of the intragastric pH than that of a PPI alone. This crossover study was designed to compare the acute effect on the intragastric pH of administration of omeprazole 20 mg alone with that of omeprazole 20 mg plus mosapride 5 mg.

## Methods

### Subjects

This was a randomized, two-way crossover study conducted on 10 healthy male volunteers, mean age 29.4 years (range 23 - 35 years), who were not users of acid suppressive medications, including antacids, H2RAs, and/or PPIs. All subjects were negative for anti- *Helicobacter pylori *(*H. pylori*) immunoglobulin G antibodies using E plate EIKEN H. *pylori *antibody (Eikenkagaku Inc, Tochigi, Japan).

### Study protocol and pH-metry

All subjects followed two study protocols in which they were given 20 mg omeprazole (Omeprale, Astrazeneka Pharmaceutical Co. Ltd, Tokyo, Japan) orally, or 20 mg omeprazole plus 5 mg mosapride (Gasmotin, Dainippon Sumitomo pharmaceutical Co. Ltd, Osaka, Japan), with the omeprazole being administered one hour after the mosapride. Intragastric pH was monitored continuously for 6 hours after each protocol. There was a washout period of at least 7 days between each protocol. The subjects fasted overnight (at least 8 hours) before each treatment protocol and during the 6 hours after ingestion of the drug or drugs, and both experiments were performed in the morning.

The pH electrode was inserted transnasally under local anesthesia and located in the body of the stomach. The gastric pH was measured at 10-second intervals by a portable pH meter fitted with an antimony pH electrode (Chemical Instrument Co. Ltd, Tokyo, Japan). The pH electrode was calibrated before each recording using standard buffers of pH 4.01 and 6.86. The pH data were analyzed using established software (Chemical Instrument Co. Ltd, Tokyo, Japan). The average pH and the percent durations over which the intragastric pH remained above 1, 2, 3, 3.5, 4, 5, 6, 7 and 8 during the 6-hour monitoring period after the administration of each drug were also measured.

### Statistics

Statistical evaluation was carried out using the Wilcoxon's signed-rank test. The level of significance was set at *p *< 0.05. All the statistical analyses were performed using the StatView program (SAS Institute, Cary, NC, USA).

### Ethics

The study was conducted in accordance with the principles of the Declaration of Helsinki, and with the approval of the Ethics Committee of Yokohama City University School of Medicine. We obtained informed consent for participation in the study from all the volunteers.

## Results

All subjects completed the study. No adverse events were recorded during the study.

### Average pH

**Figure 1:**
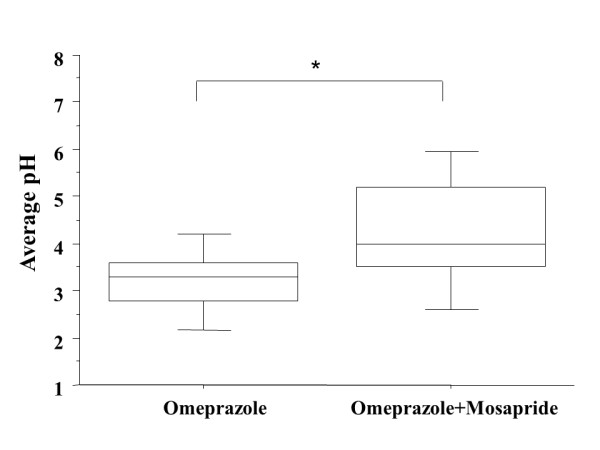
**Average pH during the first 4 hours was higher after administration of omeprazple with mosapride than after omeprazole**. *(*p *= 0.0247 by the Wilcoxon signed-ranks test).

The average pH during the 6-hour period after the administration of omeprazole 20 mg plus mosapride 5 mg was higher than that after administration of omeprazole 20 mg alone (median: 4.21 versus 3.22, respectively; *p *= 0.0247) (Figure 1).

**Figure 2:**
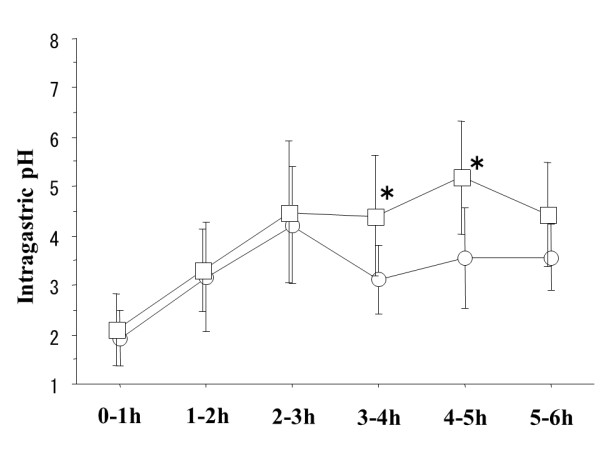
**Omeprazole 20 mg with mosapride 5 mg provided a higher average pH, compared to omeprazole 20 mg alone, at the 3-4 hour and 4-5 hour study period after administration**. Squares (omeprazole with mosapride) and circles (omeprazole), mean values; vertical lines, standard deviation (SD); horizontal line, ± SD. *p = 0.0284 and 0.0093 by the Wilcoxon signed-ranks test.

The average pH was significantly higher after administration of omeprazole 20 mg plus mosapride 5 mg than that after omeprazole 20 mg alone during the 3 to 4 and 4 to 5-hour study periods (median: 4.35 versus 2.75; *p *= 0.0284, 5.35 versus 3.45; *p *= 0.0093) (Figure 2). No significant differences were found in the 0-1, 1-2, 2-3 and 5-6 hour study periods.

### Holding time (%) of various pH levels over the 6-hour monitoring period

**Figure 3:**
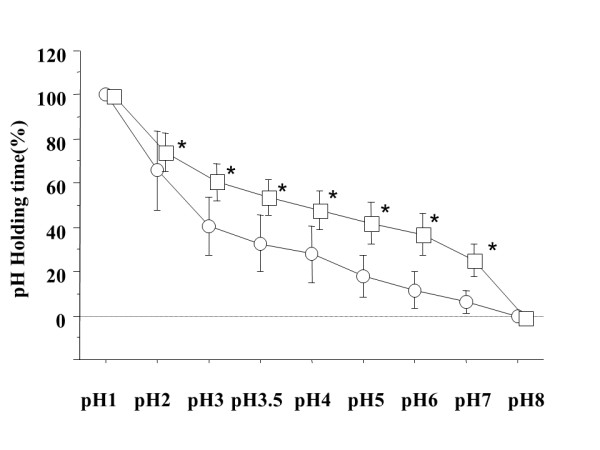
**During the 4-hour study period, omepraaole 20 mg with mosapride 5 mg provided a longer duration of pH > 3, 3.5, 4, 5, 6 and 7, compared to omeprazole 20 mg alone**. Squares (omeprazole with mosapride) and circles (omeprazole), mean values; vertical lines, standard deviation (SD); horizontal line, ± SD. *p = 0.0367, 0.0469, 0.0367, 0.0217, 0.0109 and 0.0173 by the Wilcoxon signed-ranks test.

During the 6-hour study period, administration of omeprazole 20 mg plus mosapride 5 mg provided a longer duration of pH > 3, 3.5, 4, 5, 6 and 7 as compared with that of omeprazole 20 mg alone (median: 67.6% versus 36.8%; *p *= 0.0367, 55.9% versus 34.1%; *p *= 0.0469, 49.5% versus 28.7%; *p *= 0.0367, 45.6% versus 16.8%; *p *= 0.0217, 38.6% versus 7.6%; *p *= 0.0109, and 20.4% versus 4.4%; *p *= 0.0173) (Figure 3). duration of pH > 2 compared to omeprazole 20 mg alone (median: 80.2% versus 62.6%; *p *= 0.5076)

## Discussion

In this study, we examined the change of the intragastric pH after a single oral administration of omeprazole 20 mg plus mosapride 5 mg as compared with that following administration of omeprazole 20 mg alone in the early post-administration phase in *H. pylori*-negative subjects.

Mosapride citrate (mosapride) (4-amino-5-chloro-2-ethoxy-N-{[4-(4-fluorobenzyl)-2-morpholinyl]methyl}benzamide citrate) is a novel gastrokinetic agent that enhances gastrointestinal motility by stimulating the serotonin (5-HT4) receptor [4]. Mosapride stimulates acetylcholine release from the cholinergic nerve endings in the gastrointestinal wall and may enhance upper gastrointestinal motor activity in the postprandial state in conscious dogs [5]. After oral administration, mosapride is absorbed in the small intestine in rats, rather than in the stomach [6].

Mosapride accelerates gastric emptying in healthy adults. This study may indicate that mosapride accelerates the absorption of omeprazole. For example, mosapride accelerated the gastric emptying and completion rate of small bowel examinations in patients undergoing capsule endoscopy [7]. The gastric emptying time (GET) in the mosapride group was reduced and indicated the obvious effect of mosapride on the shortening of the GET. Moreover, preparation for barium enemas using mosapride before and after oral intestinal lavage solution (PEG-ELS) intake is more effective than the modified Brown's method that is commonly used in Japan [8]. Mosapride improves gastrointestinal motility and reduces gastric stasis or gastroesophageal reflux [9,10]. Mosapride also alleviates gastrointestinal dysfunction.

Although many factors are implicated in the development of gastroesophageal reflux disease (GERD), acid reflux to the esophagus is considered to be the major cause of this disease. Treatment with PPIs to provide potent, long-term suppression of gastric acid is essential for disease management. On the other hand, the transient heartburn associated with mild GERD is attributed mainly to temporary, short-term gastric acid reflux. For example, water and antacid immediately increased the gastric pH, while PPIs showed a delayed, but prolonged effect as compared to H2RAs [11]. Therefore, rapid acid suppression is one of the most important factors for the resolution of these symptoms. Because administration of omeprazole plus mosapride promptly suppressed gastric acid secretion [12], it was originally considered to be a useful drug for on-demand treatment of mild GERD. Omeprazole used with mosapride may accelerate the onset of action, and may thus be more suitable for on-demand use than omeprazole alone. Furthermore, fixed mosapride-omeprazole combination therapy may be more useful than omeprazole alone.

If mosapride accelerates the absorption of omeprazole, why was the gastric pH not higher during 0-1, 1-2, or 2-3-hour period (Figure 2)? We suspect that mosapride has not so quick and strong effect for earlier phase of gastric emptying. If mosapride can accelerate gastric emptying quickly for 1 hour compare to control, earlier effect may be observed during 0-1 or 1-2 or 2-3 hour study period.

Intragastric pH during 3-4-hour study period was fallen (Figure 2). Why this transient decrease of intragastric pH observed using PPI? Because PPI can block only activated proton pomp, gastric pH does not reflect blood concentration of omeprazole directory. Our previous manuscripts [13-15] reported that after PPI administration, intragastric pH value raise showing up and down.

The limitations of the present study are: short time (6 hours) study period may be insufficient for the end of both mosapride's and omeprazole's effects for intragastric pH, and data were collected from healthy volunteers and not GERD patients requiring on-demand therapy. Further study may be necessary to evaluate these problems.

The ideal medication for the treatment of heartburn should have the rapid onset of action needed for on-demand treatment, as well as a sufficient duration of action to ensure that the symptoms are controlled. On the basis of our results, we conclude that omeprazole 20 mg plus mosapride 5 mg produced a rise in intragastric pH more promptly than omeprazole 20 mg alone in *H. pylori*-negative healthy male subjects. The clinical implications of our results remain unclear; however, our findings suggest that oral administration of the combination of omeprazole 20 mg preceded by mosapride 5 mg tablets may be suitable for on-demand treatment in patients with mild GERD. Further study may be necessary to evaluate the effects in GERD subjects.

## Conclusions

In conclusion, in *H. pylori-*negative healthy male subjects, an oral dose of omeprazole 20 mg plus mosapride 5 mg increased the intragastric pH more rapidly than that of omeprazole 20 mg alone.

## Abbreviations

H2RAs: H2 receptor antagonists; PPIs: Proton pump inhibitors; 5-HT4: 5-Hydroxytryptamine receptor 4; GET: Gastric emptying time; PEG-ELS: Polyethylene glycol electrolyte; GERD: Gastroesophageal reflux disease.

## Competing interests

The authors declare that they have no competing interests.

## Authors' contributions

HI analyzed, collected the clinical data and wrote the manuscript, with contributions from MI. AN was responsible for the design of the study and collected the clinical data. MI performed the statistical analyses. HI and MI analyzed the clinical data and participated in the design and coordination of the study. All authors read and approved the final manuscript.

## Pre-publication history

The pre-publication history for this paper can be accessed here:

http://www.biomedcentral.com/1471-230X/12/25/prepub
